# Measured and Modeled Toxicokinetics in Cultured Fish Cells and Application to *In Vitro* - *In Vivo* Toxicity Extrapolation

**DOI:** 10.1371/journal.pone.0092303

**Published:** 2014-03-19

**Authors:** Julita Stadnicka-Michalak, Katrin Tanneberger, Kristin Schirmer, Roman Ashauer

**Affiliations:** 1 Eawag, Swiss Federal Institute of Aquatic Science and Technology, Dübendorf, Switzerland; 2 ETH Zürich, Institute of Biogeochemistry and Pollutant Dynamics, Zürich, Switzerland; 3 EPF Lausanne, School of Architecture, Civil and Environmental Engineering, Lausanne, Switzerland; 4 Ecosens AG, Wallisellen, Switzerland; 5 Environment Department, University of York, Heslington, York, United Kingdom; Biological Research Centre of the Hungarian Academy of Sciences, Hungary

## Abstract

Effect concentrations in the toxicity assessment of chemicals with fish and fish cells are generally based on external exposure concentrations. External concentrations as dose metrics, may, however, hamper interpretation and extrapolation of toxicological effects because it is the internal concentration that gives rise to the biological effective dose. Thus, we need to understand the relationship between the external and internal concentrations of chemicals. The objectives of this study were to: (i) elucidate the time-course of the concentration of chemicals with a wide range of physicochemical properties in the compartments of an *in vitro* test system, (ii) derive a predictive model for toxicokinetics in the *in vitro* test system, (iii) test the hypothesis that internal effect concentrations in fish (*in vivo*) and fish cell lines (*in vitro*) correlate, and (iv) develop a quantitative *in vitro* to *in vivo* toxicity extrapolation method for fish acute toxicity. To achieve these goals, time-dependent amounts of organic chemicals were measured in medium, cells (RTgill-W1) and the plastic of exposure wells. Then, the relation between uptake, elimination rate constants, and log K_OW_ was investigated for cells in order to develop a toxicokinetic model. This model was used to predict internal effect concentrations in cells, which were compared with internal effect concentrations in fish gills predicted by a Physiologically Based Toxicokinetic model. Our model could predict concentrations of non-volatile organic chemicals with log K_OW_ between 0.5 and 7 in cells. The correlation of the log ratio of internal effect concentrations in fish gills and the fish gill cell line with the log K_OW_ was significant (r>0.85, p = 0.0008, F-test). This ratio can be predicted from the log K_OW_ of the chemical (77% of variance explained), comprising a promising model to predict lethal effects on fish based on *in vitro* data.

## Introduction

Environmental regulations require comprehensive testing and risk assessment before a chemical can be approved for use. In assessing the environmental risk of chemicals, fish play a very important role, being the most frequently tested vertebrate representative for freshwater systems [1]. *In vitro* fish cell assays are considered to be a promising alternative to fish bioassays to replace or reduce the use of fish in toxicological testing [2], [3]._ENREF_3 Cells in culture plates or vials can be exposed to a large number of chemicals and toxicity after exposure to chemicals can be quickly analyzed_ENREF_3. In addition, few, if any, animals are used, little test substance is needed, and little toxic waste is produced_ENREF_4. For instance, *in vitro* fish liver cell assays, using freshly isolated hepatocytes, can be applied for *in vitro – in vivo* extrapolation of chemical biotransformation in fish [4], [5]. In addition, permanent fish cell lines, which can be cultured indefinitely without further need of animals, provide another potential route for establishing *in vitro*– *in vivo* toxicity extrapolations. Tanneberger et al. [6] highlighted that, because gill epithelia are the primary uptake site of water-born contaminants into fish, they could also be a primary target for many toxicants in exposure scenarios where vital epithelial cell functions are destroyed, resulting in a toxic effect on the whole organism. Along these lines, Li et al. [7] noticed that in fish, gill tissue can be more sensitive to some chemicals than liver and muscle tissues. For these reasons, understanding the toxicokinetics in gill cells and the resulting improvement of *in vitro – in vivo* toxicity extrapolations is very important.

The quantification of chemical toxicity in cells is generally based on nominal (i.e. intended) chemical concentrations. However, recent studies show that measurements of external exposure are more appropriate than nominal concentrations due to the number of competing processes occurring in the culture well, like sorption to various compartments in a well or evaporation [3], [6], [8]. Yet, external concentrations as dose metric are still only a surrogate which may impede interpretation and extrapolation of toxicological effects because internal concentrations are thought to give rise to the biologically effective dose [9], [10]. In particular, the extrapolation of toxicity to other species, compounds and exposure patterns benefits from using dose metrics based on toxicokinetics (TK) [11], [12]. Toxicokinetics describes the time-course of a chemical concentration in a relevant biological matrix (e.g., cells in an *in vitro* assay or a tissue within the intact organism). For these reasons, we also need to understand the relationship between the external and internal concentration of chemicals in cells of *in vitro* cell line test systems. The quantification of the time course of internal concentrations in cells and whole organisms facilitates a better understanding of toxicity and may improve *in vitro* to *in vivo* toxicity extrapolation. Finally, following the tissue-residue approach, which proposes the use of tissue or total internal concentrations as the dose metric for characterizing a toxicant's potency [13]–[15], one can derive the hypothesis that, if the chemical acts by the same mode of action in cells and intact animal, the concentrations in an organism that cause toxicity must be similar to the concentrations that cause toxicity in a cell line. Support for this hypothesis was provided by research on surfactants, which elicited toxicity at fish cell residue levels corresponding closely to *in vivo* residue levels associated with surfactant toxicity [16]. The tissue residue hypothesis can be even further refined by using the free (unbound) internal concentration as dose metric. This is thought to be even closer to the biologically relevant dose metric than total internal concentrations [17], [18]; however, we here assume that a larger fraction of the uncertainty in *in vitro* to *in vivo* toxicity extrapolation originates from the difference between external and internal concentrations. Thus, we focus on improving quantification of total internal concentrations as a first step.

Thus far, existing modeling approaches for predicting chemical concentrations in cells assume equilibrium conditions [17], [19]._ENREF_16 However, it was shown, in particular for volatile and hydrophobic compounds, that obtaining stable exposure concentrations is difficult and that measured chemical concentrations in the test medium can differ significantly from nominal concentrations [8]. For this reason, partition-controlled dosing systems for *in vitro* cell assays, which allow stable exposures to be achieved, were developed [20], [21]. However, the applicability of these systems to a wide range of chemicals still needs to be addressed as only few chemicals were tested thus far and handling is still cumbersome. A TK model, which describes the chemical distribution in the experiment's environment also under non equilibrium conditions, would be a useful alternative. Such a model, which takes into account not only chemical uptake by cells but also evaporation and binding to plastic, could support quantifying and understanding the toxicity of chemicals toward cells.

Thus, the objectives of this study were as follows: (i) elucidate the time-course of the concentration of chemicals with a wide range of physicochemical properties in the compartments of an *in vitro* toxicity test system, (ii) derive a general, predictive model for toxicokinetics in the *in vitro* system, (iii) test the hypothesis that internal effect concentrations in fish (*in vivo*) and fish cell lines (*in vitro*) correlate, and (iv) develop a quantitative *in vitro* to *in vivo* toxicity extrapolation method for fish acute toxicity.

## Materials and Methods

### Study outline

A set of substances characterized by a wide range of Henry's Law Constants (log H), reflecting volatility, and by a wide range of octanol-water partition coefficients (log K_OW_), reflecting hydrophobicity, was tested as these parameters can impact the toxicity to fish and cells [22] and determine the fate of the substance in the *in vitro* test system. The amount of each chemical in medium, cells and plastic of the well plate for various time points was measured in order to study the chemicals' distribution (Figure S1 in File S1). The relation between uptake and elimination rate constants and log K_OW_ was investigated in order to develop a multi-compartment TK model describing the chemical distribution in the exposure well. In addition, based on previous *in vitro* toxicity studies [6], [23], the empirically obtained multi-compartment TK model was used to predict internal effect concentrations in cells (IEC50), which were compared with internal effect concentrations in fish gills (ILC50) predicted by a Physiologically Based Toxicokinetic (PBTK) model [24].

### Chemicals

For quantification, ^14^C-labelled chemicals were used. The applied chemical concentrations as well as the chemicals' physicochemical properties are presented in Table 1 (for radioactivity data see Table S1 in File S1). Chemical amounts were quantified based on total radioactivity. Eight substances with low volatility (log H<−6 atm·m^3^/mol) and three substances with high volatility (log H>−4 atm·m^3^/mol) were used. Stock solutions were prepared using methanol (Acros Organics, Geel, Belgium) with the exception of hexachlorobenzene (Sigma-Aldrich, Buchs, Switzerland), which was dissolved in DMSO (dimethyl sulfoxide). The final concentrations of solvents in the exposure system were 0.15% v/v for DMSO and ≤0.16% for methanol. Chemical concentrations added to the medium at the beginning of the experiments were chosen based on two criteria: to be high enough to ensure detection in test medium, cells and plastic using Liquid Scintillation Counting (LSC, Tri-Carb 2200CA, Packard, USA) and, if possible, to be below toxic levels. Chemical concentrations were considered as non-toxic if the number of cells in the exposed wells was within ±10% of the cell number in wells with no chemicals (control) for all time points as determined with the fluorescamine assay (see below). The above mentioned criteria could be fulfilled for all chemicals except for pentachlorophenol for which the limit of detection required us to use a concentration that caused death of 20–30% of cells (in agreement with Tanneberger et al. [6]).

**Table 1:** Properties and concentrations of the test chemicals used for measuring and predicting chemical concentrations in the RTgill-W1 cell line.

Chemical	CAS	Molecular weight (g/mol)	Log K_OW_ a	Log H^a^ (atm, m^3^/mol)	Concentration in medium^b^ (μg/L)
Imidacloprid	138261-41-3	255.66	0.57	−14.78	24.5
Dimethoate	60-51-5	229.26	0.78	−9.61	58.0
Carbendazim	10605-21-7	191.19	1.52	−10.67	17.5
Malathion	121-75-5	330.36	2.36	−8.31	64.0
Cyproconazole	94361-06-5	291.78	2.9	−9.15	23.5
Propiconazole	60207-90-1	342.22	3.72	−8.76	56.0
Pentachlorophenol	87-86-5	266.34	5.12	−7.61	5.0
Cypermethrin	52315-07-8	416.31	6.6	−6.38	1.3
*1,2,3-Trichlorobenzene*	87-61-6	181.45	4.05	−2.90	6.6
*Naphtalene*	91-20-3	128.18	3.3	−3.36	4.3
*Hexachlorobenzene*	118-74-1	284.78	5.73	−2.77	7.62

a - logK_ow_ and log H were taken from EPI Suite: experimental database.

b - nominal chemical concentration dosed at the beginning of the experiment

*italic font*– volatile compounds.

### Cell culture

The RTgill-W1 cell line was obtained from rainbow trout (*Oncorhynchus mykiss*) gills [25]. Details about the routine cell culture and exposure setup are available in File S1.

One ml of Leibovitz (L15) medium (LuBio Science GmbH, Luzern, Switzerland), containing 350 000 cells determined based on electric field multi-channel cell counting (CASY1 TCC, Schärfe System, Germany), was added into the wells of 24-well plates (Greiner Bio-One, Frickenhausen, Germany) in order to seed cells for exposure. The incubation time after seeding cells and prior to exposure was between 24 and 30 hours to allow cells to form a confluent monolayer containing around 400 000 cells.

### Exposure of cells to chemicals

At the beginning of the experiment and for each time point, the number of seeded cells was determined based on protein content measurements. The protein assay was chosen so that cell number could be determined directly in the cell culture wells. The rainbow trout gill cells were exposed to chemicals in 24-well tissue culture plates for 0, 1, 2, 4, 8, 16, 24 and 48 hours at 19°C in normal atmosphere. Dosing stocks were prepared in the exposure medium, L15/ex [26], in which the cells are viable but no longer proliferate, so the cell culture system remains stable. L15/ex is a modified Leibovitz medium and includes only galactose, sodium pyruvate and salts.

Cells from all well plates were washed with 1 ml of L15/ex before the chemical or control (solvent) was added. For each time point, triplicate wells were dosed in two culture plates: one plate was used to measure protein content (see “Determination of cell number”) while the second plate was sampled to measure radioactivity in each compartment and derive a mass balance. During the whole experiment, all culture plates were covered with plastic foil (VWR International GmbH, Darmstadt, Germany) in order to reduce evaporation.

### Chemical extraction

On termination of exposure, first, 100 μl of medium were taken from all respective wells and added into 20 ml glass vials, each filled with 10 ml of Ecoscint A – liquid scintillation cocktail (ChemieBrunschwig, Basel, Switzerland). Then, all of the remaining medium was removed and replaced with 100 μl of versene (LuBioScience GmbH, Luzern, Switzerland). Next, 100 μl of trypsin (ChemieBrunschwig, Basel, Switzerland) was added to these wells to detach cells. After a few minutes, the detached cells, together with versene and trypsin, were pipetted into glass vials pre-filled with 10 ml of Ecoscint A.

Two ml of methanol were added to empty wells (from which the cells and the chemical had been removed) in order to extract the chemical from plastic. Then, the wells were covered again with the same plastic foil (VWR International GmbH, Darmstadt, Germany) and the whole well plate was wrapped with aluminum foil and shaken for 10 minutes. Methanol with the extracted chemical was taken from the wells and each added into a glass vial, pre-filled with 10 ml of Hionic Fluor (liquid scintillation cocktail for organic solvents; Perkin, Elmer, Massachusetts, USA).

Details about converting chemical radioactivity to concentration are provided in File S1. For a mass balance approach, concentrations were converted to % of chemical added to the medium (three technical replicates; see Table S4 in File S1). As shown in this Table, exposures and chemical analysis were performed for two independent biological replicates (i.e. using cells from different passages) for four of the tested compounds (pentachlorophenol, malathion, propiconazole and hexachlorobenzene), giving very similar results. The other chemicals were then tested in a single experiment (as shown in Table S4 in File S1).

### Determination of cell number

The cell number in each well was determined based on total protein content of the cells using the fluorescamine assay method. Cell number was calculated based on a standard curve depicting the relationship between cell number, protein content and fluorescence (see details in Figure S2 in File S1).

For each time point, L15/ex medium was removed from wells, and cells were rinsed with 500 μl of PBS (Dulbecco's Phosphate Buffered Saline w/o calcium and magnesium). Then PBS was replaced by 500 μl of nanopure water, and the well plate was stored at −80°C for at least one hour to disrupt cells. After thawing, 1 ml of PBS and then 0.5 ml of fluorescamine (Sigma-Aldrich, Buchs, Switzerland) diluted in acetone (3 mg of fluorescamine per 10 ml of acetone), was added to each well containing cells and to one additional, cell free, well per plate as a control. Then, each plate was covered with aluminum foil and shaken for 5 minutes. The fluorescence was measured in each well using the Infinite M200 microplate reader (TECAN, Männedorf, Switzerland; excitation: 360 nm, emission: 460 nm).

### Modeling chemical distribution in a well

The time course of chemical concentrations was modeled in three compartments of the *in vitro* test system: cells, plastic and medium. For volatile compounds also a fourth compartment, headspace, was modeled. For this, uptake and elimination rate constants were fitted based on the total loss of a chemical deduced from the mass balance. The model and measurements did not include biotransformation nor transformation products at this point. The simple exposure medium L15/ex does not favor metabolic processes in general; for example, cells in this medium do not proliferate [26]. However, physiological metabolism is not necessarily correlated with the ability to biotransform xenobiotics and dedicated research, beyond the scope of this study, is needed to characterize biotransformation processes in cultured fish cells.

In each compartment, chemical concentrations were simulated based on the following equation:
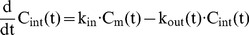
(eq.1)where C_int_(t) is the chemical concentration in the compartment, i.e. internal concentration (cells: amount × mass^−1^, plastic: amount × surface^−1^, headspace: amount × volume^−1^), C_m_(t) is the chemical concentration in the medium (amount × volume^−1^), k_in_ is the uptake rate constant (cells: volume × mass^−1^ × time^−1^, plastic: volume × surface^−1^ × time^−1^, headspace: volume × volume^−1^ × time^−1^) and k_out_ is the elimination rate constant (time^−1^).

Uptake and elimination rate constants were fitted to measured concentrations of each chemical in the respective compartment both separately and simultaneously by minimizing the sum of squares between measured and modeled concentrations using the Levenberg–Marquardt algorithm. Optimized parameter values were similar for both methods (see Table S2 in File S1). Because fitting rate constants separately for each compartment yields more accurate predictions of chemical concentrations in cells, results and discussion are presented for rate constants which were fitted by modeling concentrations in each compartment separately.

Time to steady-state conditions was calculated for each chemical, taking into account the fluctuation of chemical concentrations in medium over time, based on the elimination rate constant and assuming 90% attainment of steady-state before the compound was judged to be at steady-state [27] (see eq. S1, Table S3 in File S1).

The model was implemented and solved using ModelMaker (version 4.0, Cherwell Scientific Ltd., Oxford, UK). Details about model equations, implementation and calibration are presented in File S1.

### Internal effect concentrations of chemicals: cells and fish

Fish toxicity values, for example those taken from the U.S. EPA fathead minnow toxicity database [28], are usually expressed as lethal concentrations for 50% of the test population (LC50), where concentration refers to that measured in the exposure medium. Similarly, Tanneberger et al. [6] expressed the concentration in the test medium that causes effects in 50% of the cell population (EC50 values) based on measured concentrations. Thus, the comparison between internal effect concentrations in fish and in fish cells (ILC50 and IEC50) was performed based on LC50 and EC50 data from the CEllSens database [6]. The CEllSens database includes EC50 values measured for the RTgill-W1 cell line in the CEllSens project and LC50 values for fathead minnow, taken from the U.S. EPA fathead minnow database [28]. EC50 values were calculated based on the assumption that toxicity is a function of the chemical concentrations available during the entire 24 hours of exposure, even if the steady-state conditions may not have been reached within this time range. From this database, two groups of chemicals were excluded: polar compounds (because their partitioning behavior cannot be well characterized by means of the octanol-water partition coefficients [29]) and highly volatile compounds (due to possible experimental artifacts, see “Modeling sorption to plastic”). In addition, only chemicals with log K_OW_ between 0.5 and 7 were chosen, as the *in vitro* TK model (this study) was calibrated for chemicals with log K_OW_ between 0.58 and 6.6 (see Table 1). Based on these criteria, 13 chemicals (Table 2) were used for the *in vitro* and *in vivo* comparison based on modeled internal effect concentrations.

**Table 2:** Properties and concentrations of chemicals selected from CEllSens project.

Chemical	CAS	Mode of Action^a^	Log K_OW_ b	Log H^b^	LC50^c^ μg/L	EC50^d^ μg/L
2,2,2-Trichloroethanol	115-20-8	NPN	1.42	−6.81	298100	102100
Diethylphthalate	84-66-2	NPN	2.42	−6.21	32100	63900
Di-n-butylphthalate	84-74-2	NPN	4.5	−5.74	831.8	250
Menadione	58-27-5	reactive	2.2	−8.51	109.6	120
Dichlorophene	97-23-4	reactive	4.26	−11.94	309	50
4-Fluoroaniline	371-40-4	reactive	1.15	−5.65	16800	200000
2,4-Dinitrophenol	51-28-5	uncoupler	1.67	−7.07	13489.6	770
Pentachlorophenol	87-86-5	uncoupler	5.12	−7.61	218.8	10
Malathion	121-75-5	AChE	2.36	−8.31	14100	12900
Disulfoton	298-04-4	AChE	4.02	−5.67	4000	1900
Parathion ethyl	56-38-2	AChE	3.83	−6.53	1584.9	810
Permethrin	52645-53-1	neurotoxic	6.50	−5.73	20	3760
Lindane	58-89-9	neurotoxic	4.14	−5.29	100	6900

a - according to Russom et al.[28] _ENREF_2_ENREF_2_ENREF_2_ENREF_2; NPN: non-polar narcosis; AChE: Acetylcholine Esterase inhibition; uncoupler: uncoupler of oxidative phosphorylation.

b - logK_ow_ and log H (atm, m^3^, mol^−1^) were taken from EPI Suite: experimental database.

c - LC50 values taken from the U.S EPA fathead minnow toxicity database [28].

d - measured EC50 values for the endpoint metabolic activity of cells [6].

Internal Effect Concentrations (IEC50) in the rainbow trout gill cell line (*in vitro*) were simulated using equation (1) and the TK parameters obtained in this study. The exposure concentration (C_m_) was set to the EC50 value and the internal concentration after 24 hours was taken as the *in vitro* IEC50. Internal Lethal Concentrations (ILC50) in the fathead minnow gill compartment were calculated using the Physiologically Based Toxicokinetic (PBTK) model developed for fish by Nichols et al. [30]–[32] and adapted for fathead minnow in our previous study [24]. The exposure was set to the 96 h-LC50 concentration (taken from the U.S. EPA fathead minnow database [28]) and the concentration in gills (richly perfused tissue) after 96 hours was taken as the ILC50 (*in vivo*). Fathead minnow weight was assumed to be 1.326 g (weight corresponding to the recommended length of fathead minnow used for LC50 tests by the OECD 203 guideline [33]; for the impact of body weight on toxicokinetics in fish see [24]).

### Statistical evaluation

Trend lines in graphs were obtained by linear or polynomial (second or third order) fitting to measured data.

Coefficient of determination (R^2^) refers to the square of the coefficient between measured and modeled values, and quantifies the fraction of the variability in the data that is explained by the model (based on the FOCUS guidance document [34]).

Coefficient of correlation (r) refers to the Pearson correlation coefficient which describes the strength of a linear correlation (association) between two variables. r = −1 represents a perfect negative correlation, while r = 1 means a perfect positive correlation.

## Results and Discussion

### Experiments with cells

**Figure 1:**
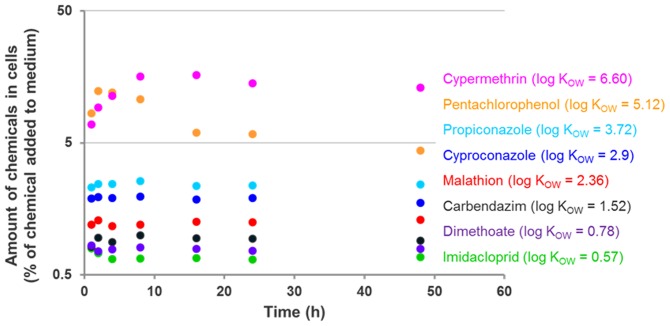
The average accumulation of chemicals in cells over time, expressed as percentage of chemical added to the medium at the beginning of the experiment. Symbols: measured values (replicates are presented in Table S4 in File S1).

For all non-volatile chemicals, mass balances were around 100% (±4% - see Table S4 in File S1). Hydrophobic chemicals (higher log K_OW_) were accumulated by cells more strongly than less hydrophobic chemicals (lower log K_OW_, Figure 1). This is consistent with the observation that hydrophobic chemicals generally accumulate more in organisms (and their cells) than hydrophilic compounds.

Figure 1 shows that amounts of chemicals characterized by low log K_OW_ values stabilized in cells within 4 hours (i.e. toxicokinetics reached equilibrium). However, due to the fact that neither cell number nor chemical concentration in medium was exactly the same at every time point, stabilization of the chemical amount in cells does not necessarily mean the stabilization of chemical concentrations in cells. Calculated times to steady-state conditions ranged from 0.6 days for compounds with low log K_OW_ to 20 days for cypermethrin (Table S3 in File S1). The 95% confidence intervals of all rate constants were reasonably small (see Table S2 in File S1) and indicate reliable calibration for all chemicals. Amounts of pentachlorophenol and cypermethrin in cells started to decrease after a few hours. For pentachlorophenol this phenomenon was attributed to its toxicity to cells. A few hours after adding pentachlorophenol to the well plates, cells started to die and detach (confirmed by microscopic observation) which resulted in the decrease of the measured total amount of chemical in the remaining cells.

**Figure 2:**
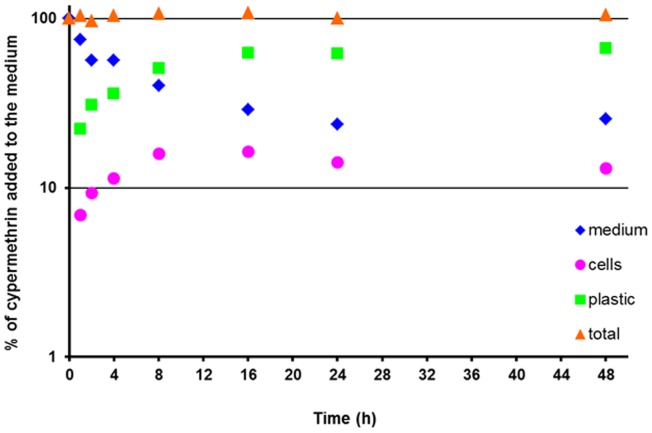
Distribution of cypermethrin in the well presented as average percentages of the chemical in each compartment over time.

Cypermethrin is very hydrophobic (log K_OW_ = 6.6) and most of the cypermethrin added to the medium was adsorbed to the plastic well (more than 50% was found in plastic and around 15% was absorbed by cells within 8 hours of exposure, Figure 2). For later time points, even though sorption of all tested chemicals to plastic was a faster process than to cells, the cypermethrin amount in the plastic was still increasing. Thus, we assume that the increase in the plastic compartment caused the decrease of the amount of cypermethrin in the medium which in turn caused the decrease of cypermethrin in the cells at late time points. This observation is in accordance with the fugacity theory which explains diffusive fluxes between matrices. In equilibrium, all matrices (compartments) have equal fugacities but until then, net fluxes occur along fugacity gradients [35].

### Modeling sorption to plastic

**Figure 3:**
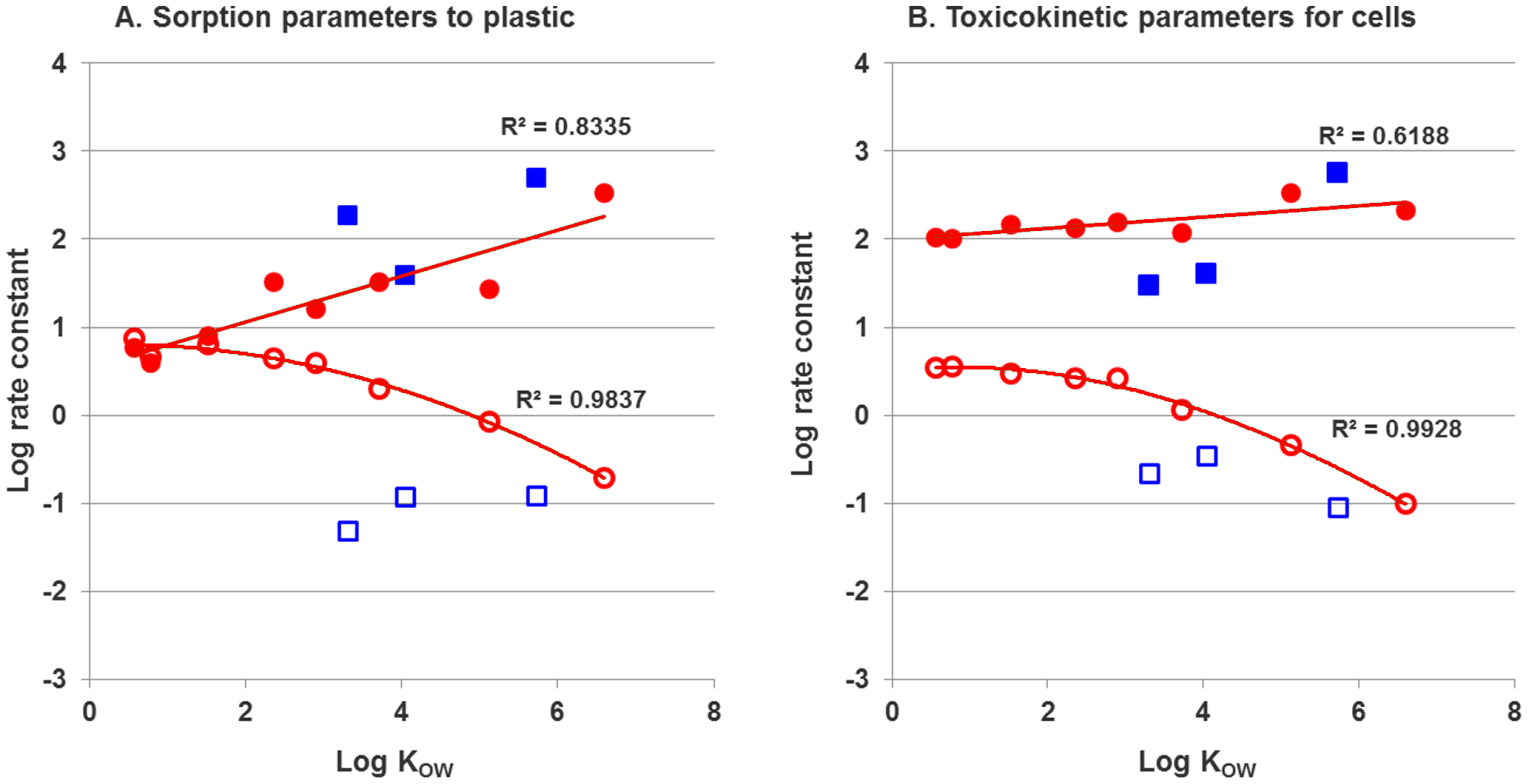
Regression of model parameters and log K_OW_ for non-volatile (red •– log k_in_, red ^O^ – log k_out_ and volatile (blue ▪ – log k_in_, blue □ – log k_out_) compounds. Data for volatile compounds were not used for fitting the model and trend lines. Red **—** – trend lines for log k_in_ and log k_out_ (A – plastic: log k_in_ = 0.2602 · log K_OW_+0.5385; log k_out_ = −0.0388 ·(log K_OW_)^2^+0.0272 · log K_OW_+0.7982, B – cells: log k_in_ = 0.0641 · log K_OW_+1.9898, log k_out_ = −0.0447 · (log K_OW_)^2^+0.0619· log K_OW_+0.525).

Uptake and elimination rate constants of non-volatile chemicals for the plastic compartment were fitted to time series of measured concentrations of each chemical in plastic. They show a strong correlation with log K_OW_ values (Figure 3A).

The relationship between logarithm of uptake rate constant (log k_in_) and log K_OW_ was linear (coefficient of determination R^2^ = 0.834) while the relationship between elimination rate constant (log k_out_) and log K_OW_ could be well described by a second-order polynomial (R^2^ = 0.984); however, exact values of parameters might differ for different test conditions. The strong binding to plastic was shown previously and it can be influenced by serum content (serum was not present in the medium used in our study) [19], [26], [36].

For volatile compounds, uptake rate constants are similar to these for non-volatiles, while elimination rate constants are much lower for volatile chemicals (Figure 3A). This results in much higher concentrations of volatile compounds in plastic (Figure S3 in File S1). One of the explanations for these differences could be related to the experimental procedures. As each culture well plate was covered with the plastic foil during the whole experiment, it is possible that some of the chemical, which had evaporated from medium, was adsorbed to that foil [37]. Then, during the shaking process to extract the chemical from plastic, methanol in the wells could have extracted the chemical also from this plastic foil which resulted in an apparent higher amount of the chemical measured in plastic. Thus, the measured distribution to the plastic and the rate constants derived from those are possibly influenced by an experimental artifact that leads to larger measurement errors for volatile compounds. We therefore excluded uptake and elimination rate constants of volatile compounds from our model and expect that measurement and modeling of the fate of volatile compounds in *in vitro* test systems requires a different test design, possibly including sampling of the headspace, use of a different foil material as cover for the well plates, reducing headspace volume or passive dosing.

### Modeling toxicokinetics in cells

**Figure 4:**
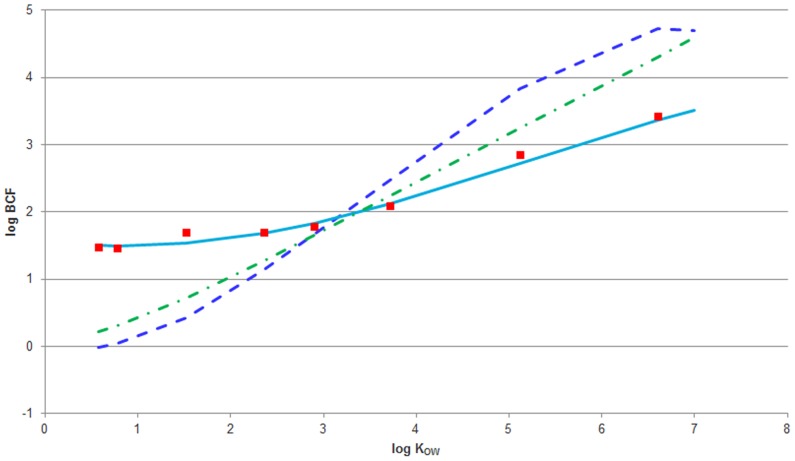
Comparison between measured and modeled bioconcentration factors (log BCF) in fish and fish cells. Average BCF measured in cells (red ▪), kinetic BCF calculated from our empirical model for rate constants (light blue **—**), equation: log BCF = −0.0078·(log K_OW_)^3^ +0.1236·(log K_OW_)^2^ −0.2073·log K_OW_+1.5872, predicted BCF in fish by the Arnot & Gobas model [38] (dark blue **- -**) and BCF predicted in rainbow trout by the PBTK model (green - •); model parameters: fish weight - 2 g, lipid fraction - 5%, water fraction - 75%). For propiconazole (log K_OW_ = 3.72) and pentachlorophenol (log K_OW_ = 5.12), steady-state condition was not reached within 24 hours.

Uptake and elimination rate constants for non-volatile compounds in cells were fitted to time series of measured concentrations of each chemical in the cells. The relationships between the *in vitro* toxicokinetic parameters and log K_OW_ (Figure 3B) were linear for log k_in_ (R^2^ = 0.619) and well described by a second-order polynomial for log k_out_ (R^2^ = 0.993). Kinetic bioconcentration factors (BCFs) were calculated as the ratio of uptake and elimination rate constants (BCF = k_in_/k_out_) and compared with measured values (BCF = internal concentration / external concentration after 24 hour exposure) and those predicted in fish by the Arnot and Gobas BCF model and by the PBTK model [38] (Figure 4).

The measured BCF value could not be derived for cypermethrin (characterized by the highest log K_OW_ value in our study) due to the decrease of this chemical amount in exposure medium over time. Furthermore, the measured BCF value for pentachlorophenol (log K_OW_ 5.12) had to be corrected for the decrease of cell number, caused by the toxicity of this chemical.

Our data and model indicate a different pattern of accumulation from what could be expected based on the assumption that the log BCF vs. log K_OW_ relationship is dominated by chemical partitioning to cell protein. According to commonly obtained chemical partitioning for tissues like plasma or muscle, which like the gill cells do not contain much lipid [39], this correlation should have a slope between 0.6–0.8 and BCF for chemicals characterized by low log K_OW_ values should be much lower than those measured in the fish cell line. To exclude the possibility of experimental artifacts caused by the influence of remaining medium on the chemical concentration in cells, additional experiments on the chemical concentration in versene and trypsine were carried out. Experiments confirmed however, that using versene and trypsin for cleaning and detaching cells did not influence chemical concentrations measured in cells.

On the other hand, common BCF models (e.g. Arnot and Gobas model) are based on lipid partitioning and log K_OW_ only. Inclusion of proteins and other tissue components could shift the fish BCF upwards by about one order of magnitude [40]. In addition, DeBruyn and Gobas [41] noticed that for chemicals characterized by low log K_OW_ values (below 2), octanol is a rather poor predictor of the sorptive capacity of proteins. Furthermore, the inaccuracy of K_OW_ and lipid based partitioning models might be greater for H-bond donor compounds [40] and based on chemical structures, all of our compounds with log K_OW_ values below 2 (i.e. imidacloprid, dimethoate and carbendazim) can be H-bond donors. For instance, the log BCF of carbendazim measured in fish varies between 1.36 and 2.2 [42], while the log BCF predicted in fish was equal to 0.42 and the log BCF measured in cells was around 1.7 (Figure 4). However, in order to understand chemical bioconcentration in fish cells better, experiments on a larger set of chemicals, characterized by an even wider range of log K_OW_ values, and with different fish cells should be carried out.

### Internal effect concentrations of chemicals: cells and fish

We used our empirically obtained toxicokinetic model to predict internal effect concentrations in cells (IEC50). These internal concentrations were then compared with internal effect concentrations in fish gills (ILC50) predicted by the Physiologically Based Toxicokinetic (PBTK) model. The basis for calculating IEC50s and ILC50s were the measured external concentrations obtained from the CEllSens project (for exact values, see Table S5 in File S1) [6], [23].

**Figure 5:**
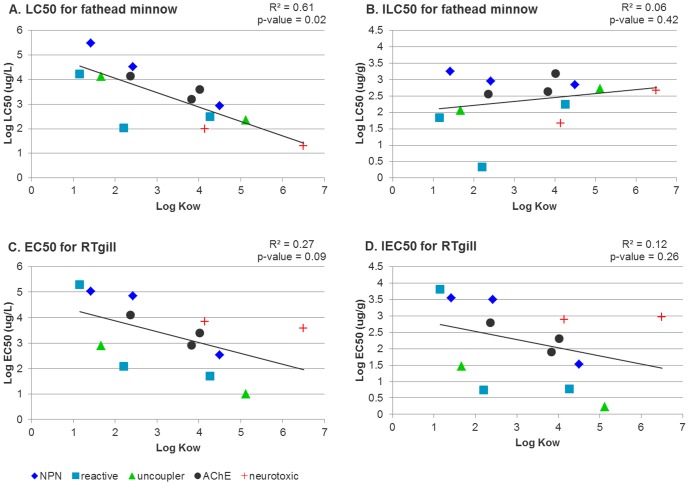
External and internal effect concentrations for fathead minnow gills (A, B) and rainbow trout gill cells (C, D). Significant correlation between effect concentrations and log K_OW_ were found only for LC50 values (log LC50 = −0.5856·log K_OW_+5.2237).

Comparing internal effect concentrations reduces a wide range of external effect concentrations (covering more than four orders of magnitude) to a narrower range of internal effect concentrations (around three orders of magnitude, Figure 5). It is important to notice that, due to the small number of data, the analysis is very sensitive to a few chemicals. However, a significant negative correlation was found between log LC50 and log K_OW_ values (Figure 5A: r = −0.78, p = 0.02, F-test), while external effect concentrations for cells (Figure 5C), as well as internal effect concentrations in fish and cells (Figure 5 B and D) were not significantly correlated with log K_OW_ (p>0.05). In addition, the lack of a relation between predicted internal effect concentrations in fish gills and a fish gill cell line (ILC50 and IEC50, see Figure S4 in File S1) did not support the hypothesis that if chemicals are characterized by the same mechanism of action in fish and fish cells, the concentrations of chemicals in a fish that cause toxicity must be similar to these concentrations in cells that cause toxicity in a fish cell line. These differences result from the pattern that chemical bioconcentration in fish is more sensitive to changes of log K_OW_ values than concentrations in cells (Figure 4). Fish BCFs increase substantially with log K_OW_. For a given toxic waterborne concentration, therefore, we can expect the internal concentration at steady-state to increase with log K_OW_. Thus, the decreasing trend in log LC50 with log K_OW_ tends to be offset by a strongly increasing trend of higher accumulation. The result is that the ILC50 is relatively constant with log K_OW_ (Figure 5B). On the other hand, for cells, BCFs do not change as much with log K_OW_ (Figure 4). For this reason, there was no significant correlation between effect concentrations in cells and log K_OW_ (Figures 5C and 5D). Our observation that internal effect concentrations do not depend on log K_OW_ - for both fish and fish cells - is in agreement with the “critical residue” approach that is used to predict waterborne concentrations likely to cause simple narcosis.

**Figure 6:**
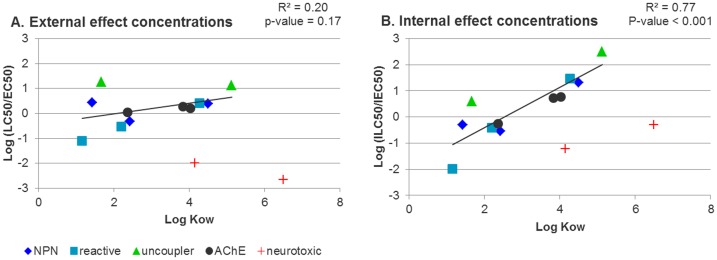
Relationship between (A) external effect concentrations in fish (LC50) and cells (EC50) vs. log K_OW_ and (B) between internal effect concentrations in fish gills (ILC50) and cells (IEC50) vs. log K_OW_. The correlation between the ratio of internal effect concentrations and log K_OW_ was significant (r = 0.86, p<0.001 F-test). Neurotoxic compounds (lindane, permethrin) are not considered in the regression line. Log (ILC50/IEC50) = 0.7736·log K_OW_−1.9537.

To further explore possible relationships between effect concentrations, we developed ratios (log LC50/EC50 and log ILC50/IEC50) that compare the effect concentrations *in vivo* and *in vitro*, and plotted these as functions of chemical log K_OW_ (Figure 6). For these calculations, lindane and permethrin were excluded from statistical analysis. These neurotoxic compounds act on sodium or chloride channels in the brain of fish, respectively, and previous studies had demonstrated that, as opposite to other chemicals from this study, their toxicity to cells [6] and even to fish embryos [43] does not reflect toxicity to fish.

The ratio between cell EC50 and fish LC50 does not depend on log K_OW_ (Figure 6A, R ^2^ = 0.2, p = 0.17, F-test), which is in accordance with Tanneberger et al. [6]. However, our analysis of internal effect concentrations revealed a significant correlation between the ratio of ILC50 and IEC50 values and log K_OW_ (Figure 6B, r = 0.86, R^2^ = 0.77, p<0.001, F-test; log (ILC50/IEC50) = 0.7736·log K_OW_ - 1.9537). This correlation could result from the fact that internal effect concentrations predicted in gills of fathead minnow and a gill cell line of rainbow trout show different patterns due to the differences in toxicokinetics between cells and organism (see Figure 5).

It is possible that the toxicity of chemicals was caused by effects in tissues other than gills; however, since most tissue “targets” reside in the well-perfused compartment, concentrations in other non-gill tissues could be expected to change in direct proportion to those in the gill. This means that even if the site of chemical action is located in tissues other than gills, it could be expected that the slopes of the log EC50 vs. log K_OW_ or log IEC50 vs. log K_OW_ for a tissue relationship are similar, although the intercepts might somewhat change. It therefore might be possible to predict effects on fish based on internal effect concentrations in cells; however, experiments on more chemicals and quantification in cells and fish tissue would be needed to either confirm or reject that hypothesis.

In conclusion, the significant correlation (r = 0.86, p<0.001, F-test) between the ratio of log ILC50 and IEC50 with log Kow values is a very promising model to predict lethal effects on fish based on *in vitro* data. Essentially, IEC50 values can be measured *in vitro* and, using the above relationship, the corresponding ILC50 value can be calculated. Then, the ILC50 value can be converted to an LC50 value using the PBTK model for fish [24]. In our study we derived an empirical prediction model so that internal concentrations in cells can now be predicted for non-volatile organic chemicals with log K_OW_ between 0.5 and 7. In the future, chemical concentrations in cells could also be linked with sublethal toxic effects, thereby providing many other potential applications in *in vitro* – *in vivo* toxicity extrapolation.

## Supporting Information

File S1 **Supporting information, figures, and tables. experimental conditions, information on chemicals, model equations and implementation.**
(DOCX)
