# Supplementary material for: Measured and Modeled Toxicokinetics in Cultured Fish Cells and Application to In Vitro - In Vivo Toxicity Extrapolation
Source: PLoS One. 2014 Mar 19;9(3):e92303. doi: 10.1371/journal.pone.0092303 (PMC3960223; doi:10.1371/journal.pone.0092303)
Supplement: File S1 — Supporting information, figures, and tables. experimental conditions, information on chemicals, model equations and implementation. (DOCX) [file pone.0092303.s001.docx]

**SUPPORTING INFORMATION**

**Measured and modeled toxicokinetics in cultured fish cells and application to *in vitro* – *in vivo* toxicity extrapolation**

Julita Stadnicka-Michalak*, Katrin Tanneberger, Kristin Schirmer, Roman Ashauer

Total page number: 13, including 5 tables and 4 figures.

[Materials and methods SI-2](#_Toc368667629)

[Chemical distribution in the well SI-2](#_Toc368667630)

[Test chemicals SI-2](#_Toc368667631)

[Cell culture SI-3](#_Toc368667632)

[Determination of cell number SI-3](#_Toc368667633)

[Converting chemical radioactivity to concentration SI-5](#_Toc368667634)

[Toxicokinetic and sorption parameters SI-5](#_Toc368667635)

[Modeled time to steady-state conditions SI-5](#_Toc368667636)

[Modeling approach SI-6](#_Toc368667637)

[Results SI-9](#_Toc368667638)

[Chemical distribution in the well SI-9](#_Toc368667639)

[Predicted values of internal effect concentrations SI-11](#_Toc368667640)

[References SI-13](#_Toc368667641)

* Corresponding author

Phone: +41 587655533

e-mail: [julita.stadnicka@gmail.com](mailto:julita.stadnicka@gmail.com)

Materials and methods

Chemical distribution in the well


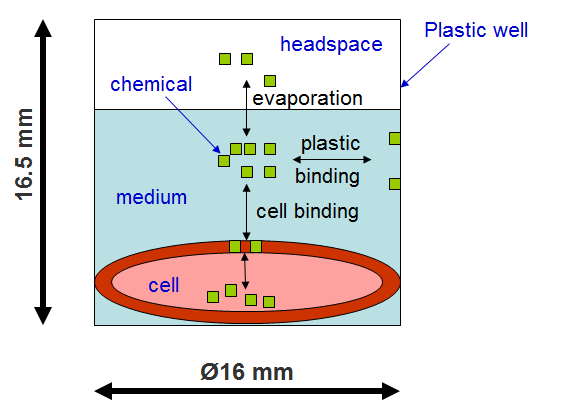


**Figure S1.** Chemical distribution in the plastic well (based on Kramer et al. [[1](#_ENREF_1)]).

Test chemicals

Toxicokinetic experiments were carried out with the following ^14^C labelled compounds: malathion ([succinyl-2,3-^14^C]-malathion, 97.78%), pentachlorophenol ([^14^C(U)], 98.05%), propiconazole ([dioxolane-4-^14^C], 99.8%), cypermethrin ([benzyl-7-^14^C], 95.23%), carbendazim (imidazole 2-^14^C], 100%), dimethoate (carbonyl-^14^C], 100%) and imidacloprid ([imidazolidine ring-14C], >95%) supplied by the Institute of Isotopes, Budapest, Hungary; 1,2,3-trichlorobenzene ([^14^C(U)], 99%), hexachlorobenzene ([^14^C(U)], 99%) and naphtalene ([^14^C(U)], >99%) supplied by American Radiolabeled Chemicals, St. Louis, USA. cyproconazole ([triazylol-U-^14^C]-cyproconazole, 98.9%) was a gift from Syngenta, Basel, Switzerland.

Unlabeled material of these compounds was of analytical grade and purchased from Sigma-Aldrich, Buchs, Switzerland.

**Table S1.** Chemical radioactivity

| **Chemical** | **Specific activity**  **(MBq/mg)** | **AMS^a^**  **(kBq/ml)** |
| --- | --- | --- |
| Imidacloprid | 4.1 | 165.4 |
| Dimethoate | 1.07 | 101.0 |
| Carbendazim | 2.87 | 73.4 |
| Malathion | 2.75 | 233.1 |
| Cyproconazole | 2.061 | 483.9 |
| Propiconazole | 2.46 | 96.06 |
| Pentachlorophenol | 1.96 | 97.14 |
| Cypermethrin | 2.83 | 89.9 |
| *1,2,3-Trichlorobenzene* | 11.62 | 499.1 |
| *Naphtalene* | 15.87 | 176.9 |
| *Hexachlorobenzene* | 10.39 | 68.90 |

^a^ - Activity in Main Stock

Cell culture

The RTgill-W1 cell line used in our study was obtained from gills of rainbow trout (*Oncorhynchus mykiss*). The RTgill-W1 cells were routinely cultured at 19°C in 75cm^2^ cell culture flasks with L15 culture medium supplemented with 5% FBS and 1% penicillin-streptomycin solution (10 mg/mL; Bioswisstec AG, Schaffhausen, Switzerland). For exposure to test chemicals, confluent cells were washed twice with versene, detached with trypsine (0.25% in PBS w/o calcium and magnesium) and re-suspended in L15 culture medium. Cells in the resulting suspension were counted using the electric field multi-channel cell counting system (CASY1 TCC, Schärfe System, Germany). Finally, 1 mL of a cell suspension with a density of 350 000 cells/mL of cell culture medium was seeded into 24-well culture plates. After 24 – 30 hours of attachment, the cells were used for testing.

Determination of cell number

It was not possible to use an electric field multi-channel cell counting system or a counting chamber in order to estimate the cell number in each well during the experiment. These methods require detaching cells which in turn may cause a decrease of cell numbers. Thus, to determine the cell number in a well, the relationship between cell number, protein content and fluorescence values was used. To obtain this relationship, the following experiments were carried out four times on four different days and using different cell passage numbers. Six different cell concentrations (nominal 400 000, 350 000, 300 000, 250 000, 200 000 and 150 000 cells per ml) were determined by using the electric field multi-channel cell counting system. Next, three replicates with one ml of each concentration were seeded in a 24-well plate. After 24h, which were required for the cells to attach, the Leibovitz medium was changed to L15/ex. The fluorescence in a well containing cells and fluorescamine (3 mg of fluorescamine per 10 ml of acetone; Sigma-Aldrich, Buchs, Switzerland) was measured after 24 and 48 hours by using the Infinite M200 microplate reader (TECAN, excitation: 360 nm, emission: 460 nm). Fluorescence values were converted to protein content based on a protein standard curve.

The protein standard curve was created by measuring values of fluorescence of fluorescamine in a control and seven different concentrations of albumin (Bovine Serum Albumin Standard, Perbio Science, Switzerland): 2, 1, 0.5, 0.25, 0.125, 0.0625 and 0.03125 mg of protein per mL.


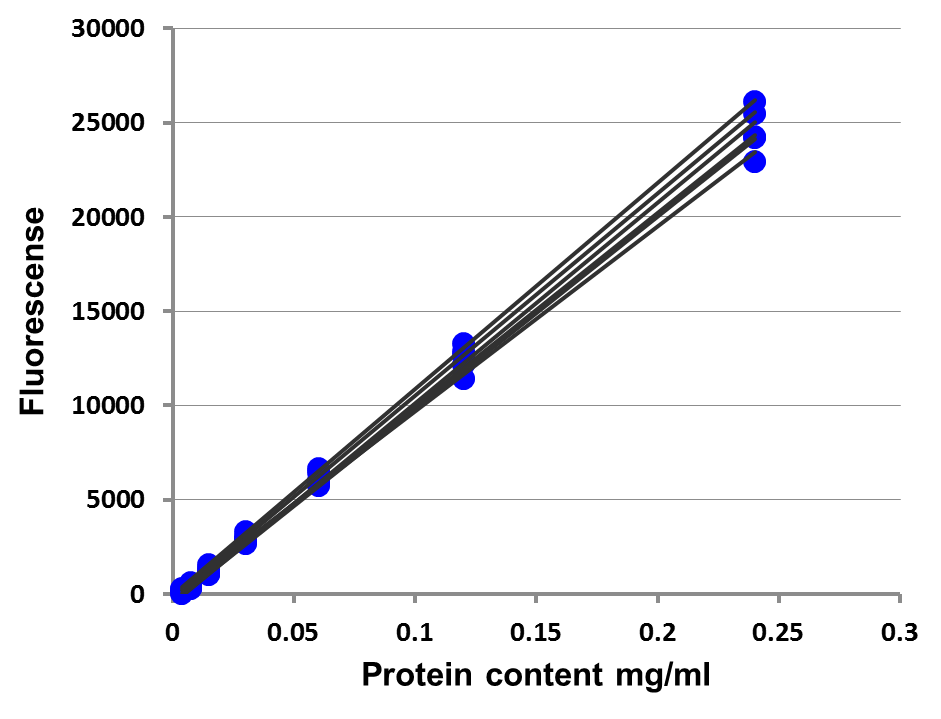


**Figure S2.** The protein standard curves created to determine the cell number (6 independent experiments, each with 3 replicates).

Converting chemical radioactivity to concentration

Chemical concentrations were obtained by converting measured radioactivity (dpm, Bq) to chemical amounts (µg) by using the specific activity of each chemical (kBq/µg) and the chemical concentrations (µg/L, µg/g, µg/cm^2^) based on compartment volume (medium – 2 mL), mass (0.000707 g wet weight calculated from the average cell number at the beginning of each experiment - 400 000 cells, average cell diameter -15 µm and density: 1g/cm^3^; equals 1.77 ng/cell) or surface (plastic surface exposed to 2 mL of medium – 5.04 cm^2^; which comprises the culture surface of the well plate).

Toxicokinetic and sorption parameters

**Table S2.** TK (for cells) and sorption (for plastic) parameters fitted separately and together. 95% confidence intervals are shown in brackets (calculated from asymptotic standard errors) [[2](#_ENREF_2)].

| Chemical | TK and sorption parameters fitted separately | | | | TK and sorption parameters fitted together | | | |
| --- | --- | --- | --- | --- | --- | --- | --- | --- |
|  | Cells | | Plastic | | Cells | | Plastic | |
|  | k_in_  L/(kg·d) | k_out_  1/d | k_in_  cm^2^/(kg·d) | k_out_  1/d | k_in_  L/(kg·d) | k_out_  1/d | k_in_  cm^2^/(kg·d) | k_out_  1/d |
| Imidacloprid | 104  (±22.1) | 3.50  (±0.89) | 5.91  (±0.23) | 7.58  (±0.71) | 105  (±29.1) | 3.50  (±0.98) | 5.92  (±1.09) | 7.62  (±1.47) |
| Dimethoate | 103  (±15.7) | 3.62  (±0.81) | 3.90  (±0.15) | 4.61  (±0.05) | 98.15  (±10.1) | 3.41  (±0.98) | 3.90  (±0.21) | 4.61  (±0.10) |
| Carbendazim | 145  (±28.9) | 2.95  (±0.526) | 7.84  (±1.49) | 6.50  (±0.18) | 90.6  (±15.86) | 2.75  (±0.496) | 4.94  (±1.56) | 3.77  (±0.39) |
| Malathion | 133  (±19.3) | 2.68  (±0.298) | 32.4  (±0.87) | 4.39  (±0.25) | 156  (±30.2) | 3.18  (±0.91) | 32.3  (±1.23) | 4.39  (±0.31) |
| Cyproconazole | 156  (±28.6) | 2.65  (±0.426) | 16.2  (±2.15) | 3.89  (±0.14) | 155  (±6.20) | 2.58  (±0.083) | 48.1  (±9.81) | 3.89  (±0.13) |
| Propiconazole | 118  (±12.5) | 1.17  (±0.138) | 32.1  (±1.87) | 2.00  (±0.11) | 116  (±7.51) | 1.10  (±0.077) | 35.1  (±3.43) | 1.96  (±0.35) |
| Pentachlorophenol | 331  (±41.4) | 0.464  (±0.05) | 27.4  (±1.98) | 0.859  (±0.06) | 283  (±24.0) | 0.252  (±0.026) | 27.4  (±2.13) | 0.861  (±0.22) |
| Cypermethrin | 188  (±27.9) | 0.116  (±0.016) | 331  (±22.1) | 0.194  (±0.02) | 239.63  (±8.44) | 0.114  (±0.006) | 220  (±36.3) | 0.088  (±0.11) |

Modeled time to steady-state conditions

Time to steady-state conditions was determined with the following equation and assuming 90% attainment of steady-state before the compound was judged to be at steady-state [[3](#_ENREF_3)]:

$\text{t} \text{(90\%)}=-\frac{\text{ln(0.1)}}{\text{k}_{\text{out}}}$, **(eq. S1)**

**Table S3.** Average time to steady-state conditions for each compound (based on Table S2).

| **Chemical** | **t_cells_ (90%), d** | **t_plastic_ (90%), d** |
| --- | --- | --- |
| Imidacloprid | 0.66 | 0.30 |
| Dimethoate | 0.64 | 0.50 |
| Carbendazim | 0.78 | 0.35 |
| Malathion | 0.86 | 0.52 |
| Cyproconazole | 0.87 | 0.59 |
| Propiconazole | 1.97 | 1.15 |
| Pentachlorophenol | 4.96 | 2.68 |
| Cypermethrin | 19.8 | 11.9 |

Modeling approach

Model runs

All used toxicokinetic models were run in **ModelMaker 4** with the following settings:

a) Run

- start value: 0,

- stop value: last day of exposure,

- repeated run: no.

b) Integration

- random seed: 1,

- integration method: Runge-Kutta,

- output points: user defined (dependent on stop value)

- fixed step: no,

- accuracy: 10^-6^,

- minimum value: 10^-10^,

- approx no of steps: 100,

- error scaling: a constant value (10).

Optimization

TK and sorption parameters of our empirically obtained model were optimized in ModelMaker 4 with the following initial settings:

1. Optimization run:

- Method: Marquardt,
- Weighting: ordinary least squares.

1. Optimization Settings:

- Convergence Change: 0.1,
- Convergence Steps: 50,
- Retry Count: 50,

1. Marquardt settings:

- Initial Lambda: 100,
- Minimum Change: 1e-200,
- Fractional Change: 0.01

Code example

compartment: Ac Unconditional

dAc/dt = (kin_cell*Am/Vm*Mc-kout_cell*Ac)

Initial Value = 0.0

compartment: Am Unconditional

dAm/dt = -kin_cell*Am/Vm*Vc+kout_cell*Ac-kin_plastic*Am+kout_plastic*Ap

Initial Value = C0*Vm

compartment: Ap Unconditional

dAp/dt = (kin_plastic*Am-kout_plastic*Ap)

Initial Value = 0.0

variable: Cc_ug_g Unconditional

Cc_ug_g = Ac/Vc

variable: Cm Unconditional

Cm = Am/Vm

variable: Cp Unconditional

Cp = Ap/Sp

Where:

Ac – chemical amount in cells, µg,

kin_cell – uptake rate constant for cells, L/(g·d),

Am – chemical amount in medium, µg,

Vm – volume of medium, L,

Mc – mass of cells, g,

kout_cell – elimination rate constant for cells, 1/d,

kin_plastic – uptake rate constant for plastic, cm^2^/(g·d),

kout_plastic – elimination rate constant for plastic, 1/d,

Ap – chemical amount in plastic, µg,

C0 – chemical concentration in medium at time point 0, µg/L,

Cc_ug_g – chemical concentration in cells, µg/g,

Cm – chemical concentration in medium, µg/L,

Cp – chemical concentration in plastic, µg/cm^2^,

Sp – plastic surface, cm^2^.

Results

Chemical distribution in the well

For four tested compounds (penatchlorophenol, malathion, propiconazole and hexachlorobenzene), chemical analysis was performed for two independent replicates.

**Table S4.** Distribution of chemicals in the well.

| Time  (h) | Pentachlorophenol (4.78 µg/L)^a^ | | | Pentachlorophenol (5.07 µg/L)^a^ | | |
| --- | --- | --- | --- | --- | --- | --- |
|  | Medium  (%) | Cells  (%) | Plastic  (%) | Medium  (%) | Cells  (%) | Plastic  (%) |
| 0 | 100 | 0 | 0 | 100 | 0 | 0 |
| 1 | 97.14 | 8.37 | 1.96 | 86.06 | 12.78 | 2.77 |
| 2 | 90.03 | 12.36 | 2.34 | 83.93 | 12.71 | 2.69 |
| 4 | 82.51 | 12.06 | 1.95 | 84.43 | 11.74 | 2.62 |
| 8 | 84.04 | 10.7 | 2.33 | 82.47 | 11.02 | 2.56 |
| 16 | 89.86 | 5.97 | 1.46 | 88.1 | 8.57 | 1.79 |
| 24 | 91.89 | 5.81 | 1.35 | 90.14 | 7.75 | 2.23 |
| 48 | 94.06 | 4.36 | 1.17 | 89.2 | 6.23 | 1.98 |

| Time  (h) | Malathion (64.55 µg/L)^a^ | | | Malathion (63.96 µg/L)^a^ | | |
| --- | --- | --- | --- | --- | --- | --- |
|  | Medium  (%) | Cells  (%) | Plastic  (%) | Medium  (%) | Cells  (%) | Plastic  (%) |
| 0 | 100 | 0 | 0 | 100 | 0 | 0 |
| 1 | 99.37 | 1.22 | 0.58 | 98.43 | 1.18 | 0.53 |
| 2 | 98.82 | 1.32 | 0.69 | 98.42 | 1.20 | 0.65 |
| 4 | 101.22 | 1.13 | 0.72 | 99.04 | 1.21 | 0.73 |
| 8 | 98.61 | 1.21 | 0.79 | 97.35 | 1.20 | 0.83 |
| 16 | 99.99 | 1.26 | 0.86 | 71.27 | 0.90 | 0.61 |
| 24 | 101.12 | 1.29 | 1.14 | 101.56 | 1.23 | 1.18 |
| 48 | 100.04 | 1.33 | 1.13 | 101.53 | 1.26 | 1.05 |

| Time  (h) | Propiconazole (56.92 µg/L)^a^ | | | Propiconazole (55.52 µg/L)^a^ | | |
| --- | --- | --- | --- | --- | --- | --- |
|  | Medium  (%) | Cells  (%) | Plastic  (%) | Medium  (%) | Cells  (%) | Plastic  (%) |
| 0 | 100 | 0 | 0 | 100 | 0 | 0 |
| 1 | 94.88 | 2.3 | 1.14 | 97.78 | 2.3 | 1.23 |
| 2 | 96.16 | 2.53 | 1.17 | 96.83 | 2.44 | 1.28 |
| 4 | 97.01 | 2.65 | 1.48 | 94.1 | 2.45 | 1.19 |
| 8 | 94.49 | 2.57 | 1.81 | 94.6 | 2.57 | 1.37 |
| 16 | 96.11 | 2.4 | 1.67 | 94.76 | 2.37 | 1.47 |
| 24 | 97.32 | 2.36 | 1.54 | 94.75 | 2.37 | 1.66 |
| 48 | 92.53 | 2.49 | 2.39 | 96.48 | 2.43 | 2 |

| Time  (h) | Hexachlorobenzene (3.70 µg/L)^a^ | | | Hexachlorobenzene (2.87 µg/L)^a^ | | |
| --- | --- | --- | --- | --- | --- | --- |
|  | Medium  (%) | Cells  (%) | Plastic  (%) | Medium  (%) | Cells  (%) | Plastic  (%) |
| 0 | 100 | 0 | 0 | 100 | 0 | 0 |
| 1 | 61.07 | 14.01 | 17.72 | 52.82 | 19.64 | 17.03 |
| 2 | 42.93 | 20.88 | 34.26 | 41.88 | 31.03 | 26.05 |
| 4 | 21.16 | 28.82 | 40.30 | 18.64 | 23.79 | 32.85 |
| 8 | 13.10 | 24.60 | 47.17 | 13.62 | 32.71 | 37.86 |
| 16 | 12.66 | 21.89 | 49.51 | 2.33 | 21.55 | 44.28 |
| 24 | 7.93 | 15.37 | 52.10 | 1.59 | 19.91 | 55.61 |
| 48 | 1.39 | 7.33 | 56.77 | 0.81 | 10.95 | 50.00 |

| Time  (h) | Cypermethrin (1.27 µg/L)^a^ | | | 1,2,3-Trichlorobenzene (2.96 µg/L)^a^ | | |
| --- | --- | --- | --- | --- | --- | --- |
|  | Medium  (%) | Cells  (%) | Plastic  (%) | Medium  (%) | Cells  (%) | Plastic  (%) |
| 0 | 100 | 0 | 0 | 100 | 0 | 0 |
| 1 | 75.22 | 6.9 | 22.21 | 57.77 | 0.65 | 10.55 |
| 2 | 56.76 | 9.3 | 30.7 | 50.34 | 0.75 | 14.88 |
| 4 | 56.44 | 11.33 | 36.1 | 26.36 | 0.51 | 17.64 |
| 8 | 40.2 | 15.95 | 50.66 | 18.2 | 0.52 | 20.25 |
| 16 | 28.94 | 16.26 | 62.81 | 9.98 | 0.41 | 17.45 |
| 24 | 23.79 | 14.13 | 61.98 | 6.33 | 0.28 | 11.38 |
| 48 | 25.36 | 13.05 | 66.88 | 4.04 | 0.21 | 7.33 |

| Time  (h) | Naphthalene (1.64µg/L)^a^ | | | Imidachloprid (24.69 µg/L)^a^ | | |
| --- | --- | --- | --- | --- | --- | --- |
|  | Medium  (%) | Cells  (%) | Plastic  (%) | Medium  (%) | Cells  (%) | Plastic  (%) |
| 0 | 100 | 0 | 0 | 100 | 0 | 0 |
| 1 | 60.25 | 0.52 | 4.54 | 99.94 | 0.79 | 0.086 |
| 2 | 50.38 | 0.51 | 6.32 | 100.85 | 0.73 | 0.068 |
| 4 | 30.40 | 0.65 | 17.65 | 100.95 | 0.66 | 0.060 |
| 8 | 19.00 | 0.56 | 28.94 | 100.83 | 0.67 | 0.075 |
| 16 | 15.09 | 0.46 | 27.61 | 98.83 | 0.67 | 0.085 |
| 24 | 13.06 | 0.41 | 25.00 | 98.25 | 0.66 | 0.069 |
| 48 | 10.83 | 0.36 | 24.21 | 100.55 | 0.68 | 0.070 |

| Time  (h) | Dimethoate (57.61 µg/L)^a^ | | | Carbendazim (17.78 µg/L)^a^ | | |
| --- | --- | --- | --- | --- | --- | --- |
|  | Medium  (%) | Cells  (%) | Plastic  (%) | Medium  (%) | Cells  (%) | Plastic  (%) |
| 0 | 100 | 0 | 0 | 100 | 0 | 0 |
| 1 | 99.85 | 0.84 | 0.060 | 100.53 | 0.82 | 0.12 |
| 2 | 100.29 | 0.76 | 0.053 | 98.91 | 0.96 | 0.12 |
| 4 | 101.16 | 0.78 | 0.051 | 97.39 | 0.89 | 0.12 |
| 8 | 101.39 | 0.81 | 0.066 | 98.77 | 1.00 | 0.13 |
| 16 | 100.70 | 0.79 | 0.060 | 100.52 | 0.95 | 0.11 |
| 24 | 99.56 | 0.76 | 0.082 | 99.36 | 0.94 | 0.14 |
| 48 | 100.61 | 0.79 | 0.068 | 100.27 | 0.78 | 0.13 |

| Time  (h) | Cyproconazole (23.51 µg/L)^a^ | | |
| --- | --- | --- | --- |
|  | Medium  (%) | Cells  (%) | Plastic  (%) |
| 0 | 100 | 0 | 0 |
| 1 | 98.51 | 1.89 | 0.33 |
| 2 | 99.67 | 1.95 | 0.38 |
| 4 | 98.77 | 1.91 | 0.43 |
| 8 | 99.30 | 1.96 | 0.40 |
| 16 | 97.51 | 1.86 | 0.41 |
| 24 | 100.39 | 1.91 | 0.45 |
| 48 | 99.95 | 1.75 | 0.44 |

^a^ – chemical concentration measured in the medium at time point 0 (just after dosing).

Predicted values of internal effect concentrations

**Table S5.** Predicted values of internal effect concentrations.

| **Chemicals** | **ILC50, µg/g** | **IEC50, µg/g** |
| --- | --- | --- |
| 2,2,2-Trichloroethanol | 1745 | 3559 |
| Diethylphthalate | 881 | 3188 |
| Di-n-butylphthalate | 699 | 32.8 |
| Menadione | 2.12 | 5.46 |
| Dichlorophene | 174 | 5.91 |
| 4-Fluoroaniline | 67.5 | 6507 |
| 2,4-Dinitrophenol | 114 | 28.9 |
| Pentachlorophenol | 514 | 1.66 |
| Malathion | 352 | 627 |
| Disulfoton | 1518 | 202 |
| Parathion ethyl | 439 | 78.6 |
| Permethrin | 462 | 908 |
| Lindane | 46.3 | 773 |

volatile

chemicals

**Figure S3.** Mass balance for chemical distribution in a well after 24 h exposure.

**
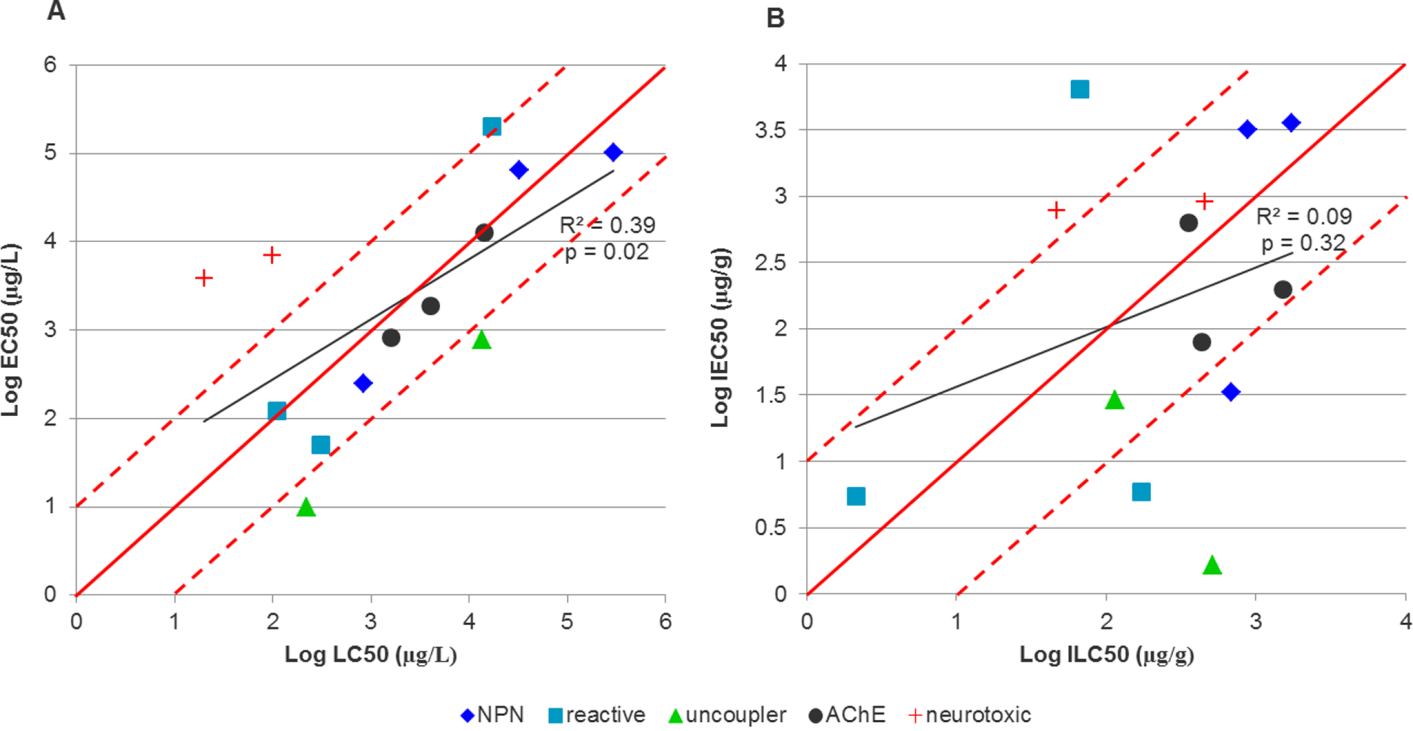
**

**Figure S4.** Correlation between external (A) or internal (B) effect concentrations for fish and fish cells. Red lines indicate the same values for fish gills and fish gill cells (line of unity) ± one order of magnitude deviation from this line(dashed lines).

References

1. Kramer NI, Krismartina M, Rico-Rico A, Blaauboer BJ, Hermens JLM (2012) Quantifying processes determining the free concentration of phenanthrene in basal cytotoxicity assays. Chemical Research in Toxicology 25: 436-445.

2. Motulsky H, Christopoulos A (2003) Fitting models to biological data using linear and nonlinear regression. A practical guide to curve fitting. GraphPad Software Inc CA, USA.

3. Hoffman D, Kringle R, Lockwood G, Turpault S, Yow E, et al. (2005) Nonlinear mixed effects modelling for estimation of steady state attainment. Pharmaceutical Statistics 4: 15-24.
